# New Insights into Therapy-Induced Progression of Cancer

**DOI:** 10.3390/ijms21217872

**Published:** 2020-10-23

**Authors:** Polina V. Shnaider, Olga M. Ivanova, Irina K. Malyants, Ksenia S. Anufrieva, Ilya A. Semenov, Marat S. Pavlyukov, Maria A. Lagarkova, Vadim M. Govorun, Victoria O. Shender

**Affiliations:** 1 Center for Precision Genome Editing and Genetic Technologies for Biomedicine, Federal Research and Clinical Center of Physical-Chemical Medicine of Federal Medical Biological Agency, Moscow 119435, Russia; polya.shnaider@yandex.ru (P.V.S.); sci.olga.ivanova@gmail.com (O.M.I.); anufrieva@phystech.edu (K.S.A.); lagar@rcpcm.org (M.A.L.); 2 Laboratory of Cell Biology, Federal Research and Clinical Center of Physical-Chemical Medicine of the Federal Medical and Biological Agency, Moscow 119435, Russia; iricam@mail.ru (I.K.M.); semenoff.ilya514@ya.ru (I.A.S.); 3 Faculty of Biology, Lomonosov Moscow State University, Moscow 119991, Russia; 4 Faculty of Chemical-Pharmaceutical Technologies and Biomedical Drugs, Mendeleev University of Chemical Technology of Russia, Moscow 125047, Russia; 5 Moscow Institute of Physics and Technology (State University), Dolgoprudny 141701, Russia; 6 Laboratory of Membrane Bioenergetics, Shemyakin-Ovchinnikov Institute of Bioorganic Chemistry of the Russian Academy of Sciences, Moscow 117997, Russia; marat.pav@mail.ru; 7 Laboratory of Simple Systems, Federal Research and Clinical Center of Physical-Chemical Medicine of the Federal Medical and Biological Agency, Moscow 119435, Russia; vgovorun@yandex.ru; 8 Laboratory of Molecular Oncology, Shemyakin-Ovchinnikov Institute of Bioorganic Chemistry of the Russian Academy of Sciences, Moscow 117997, Russia

**Keywords:** cancer progression, chemotherapy, chemoresistance, tumor microenvironment, intercellular communication, cell cycle, EMT, autophagy

## Abstract

The malignant tumor is a complex heterogeneous set of cells functioning in a no less heterogeneous microenvironment. Like any dynamic system, cancerous tumors evolve and undergo changes in response to external influences, including therapy. Initially, most tumors are susceptible to treatment. However, remaining cancer cells may rapidly reestablish the tumor after a temporary remission. These new populations of malignant cells usually have increased resistance not only to the first-line agent, but also to the second- and third-line drugs, leading to a significant decrease in patient survival. Multiple studies describe the mechanism of acquired therapy resistance. In past decades, it became clear that, in addition to the simple selection of pre-existing resistant clones, therapy induces a highly complicated and tightly regulated molecular response that allows tumors to adapt to current and even subsequent therapeutic interventions. This review summarizes mechanisms of acquired resistance, such as secondary genetic alterations, impaired function of drug transporters, and autophagy. Moreover, we describe less obvious molecular aspects of therapy resistance in cancers, including epithelial-to-mesenchymal transition, cell cycle alterations, and the role of intercellular communication. Understanding these molecular mechanisms will be beneficial in finding novel therapeutic approaches for cancer therapy.

## 1. Introduction

Due to existing advances in the development of anticancer drugs, most types of malignant tumors are susceptible to treatment. However, the results of patient treatment are not satisfying due to the development of metastasis and emergence of cancer cell resistance to applied therapy. Both of these processes often occur simultaneously [1,2,3]. The process of metastasis has been described in sufficient detail in earlier reviews [4,5]. Here we will focus our attention on how the therapy resistance emerges in the original tumors. Research shows that the incidence of recurrence varies greatly depending on the type of tumor [6]. Thus, glioblastoma relapses in nearly all patients, progression-free survival time for such patients is 2–7 months [7,8]. Similarly, in ovarian cancer patients, the relapse rate is about 85% with progression-free survival time less than two years [9,10]. Oncological diseases with a high relapse rate also include peripheral T-cell lymphoma, urinary bladder cancer, and soft tissue sarcoma [6]. On the other hand, tumors such as estrogen receptor-positive breast cancer, or kidney cancer, respond well to treatment and recur only in a small percentage of cases [11,12]. We assume that some types of malignant neoplasms possess the higher activity of certain signaling pathways or that the specific cellular organization of the tumor may allow cancers to develop resistance to treatment.

A tumor is a heterogeneous population of cells that changes significantly after exposure to drug therapy. Also, the tumor cells are in constant dynamic interaction with stromal and immune cells, as well as with other subpopulations of cancer cells. The therapeutic effect not only creates selective pressure but also changes the nature of intercellular communication, providing conditions for better survival of the remaining tumor cells [13,14]. In this review, we take a detailed look at the complex interplay of different processes that are activated in tumors and their microenvironment in response to chemo- and radiotherapy.

**Figure 1:**
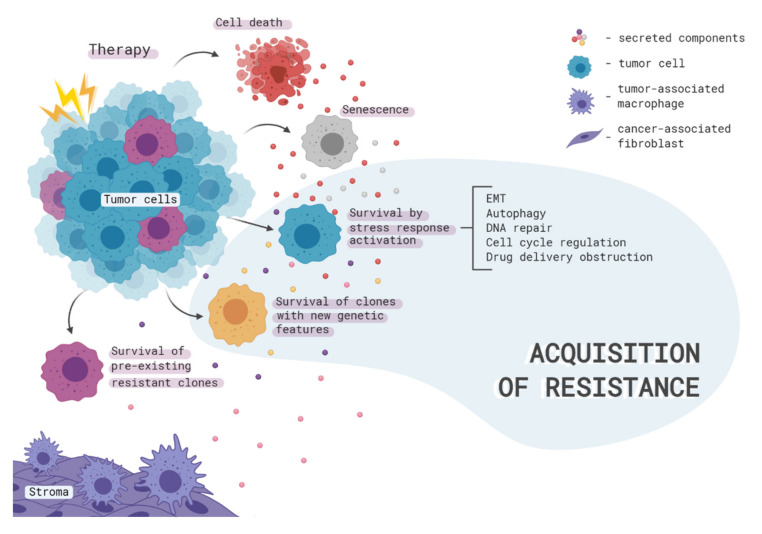
Acquisition of cancer cell resistance from the population point of view. The tumor is heterogeneous and consists of both therapy-sensitive and resistant cancer cells. Also, tumor-associated immune and stromal cells surround the tumor, creating a unique tumor microenvironment. Therapy simultaneously triggers many events: cell death, the transition to a state of senescence, the survival of pre-existing resistant clones, and the acquisition of new genetic and epigenetic features by cells, as well as activation of stress response cascades therein. All these processes lead not only to a change in the cellular composition of the tumor and tumor stroma but also to the transformation of the secretion profiles of all participants in intercellular communication. Such communication enhances the efficiency of the cellular response to stress, which, together with genomic instability and clonal selection, ensures the adaptation of cells to therapy, expansion of the most resistant tumor populations, and tumor recurrence.

**Figure 2:**
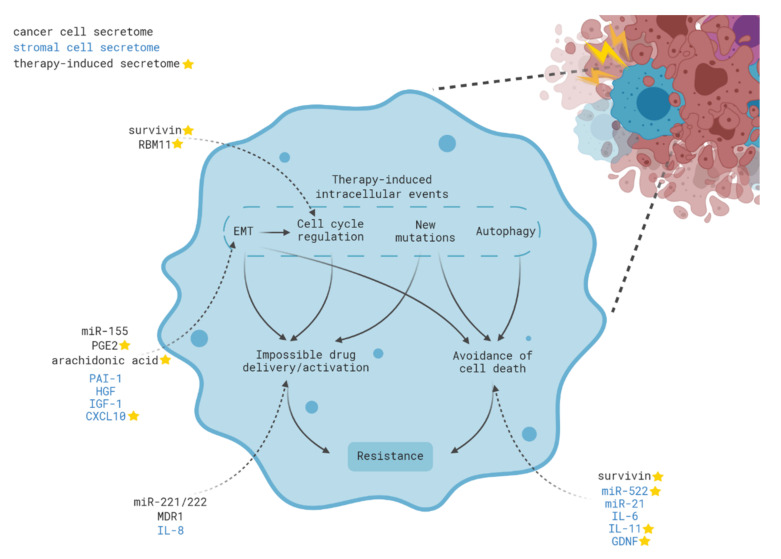
The role of the therapy-induced intra- and intercellular events in the acquisition of cancer cell resistance. Stress caused by anticancer therapy triggers many events (acquisition of new mutations, changes in the regulation of the cell cycle, EMT, autophagy) which then leads to avoidance of cell death and a change in the efficiency of absorption/activation of the chemotherapy drug. In addition to intracellular signals, molecules from stromal cells, as well as other tumor cells, entering the cell from outside are involved in the induction of these events. Molecules of therapy-induced secretomes are marked by asterisks; molecules secreted from cancer cells are marked in black; molecules secreted from stromal cells are marked in blue.

Mechanisms of acquired resistance can be subdivided based on the initial effect of therapy on cancer cells (Figure 1). In the simplest case, treatment can lead to the death of a large population of drug-sensitive cells and subsequent proliferation of pre-existing resistant cells. This mechanism is termed clonal selection. Secondly, a therapy induces temporary or permanent activation of various signaling pathways inside tumor cells enabling them to survive during treatment. Finally, the dying tumor cells can release a range of extracellular signals that trigger the formation of various mechanisms of resistance in neighboring cells (Figure 1 and Figure 2). However, it should be noted that, in a real tumor, in most cases, all three processes occur in parallel and are closely related to each other.

## 2. Therapy-Induced Clonal Selection

Modern techniques for the genome sequencing of small populations of cells located in different tumor zones [15,16,17,18,19], as well as single-cell sequencing, have enabled tracing tumor heterogeneity in vivo before and after therapy [20,21,22,23,24]. Moreover, the molecular barcoding approach made it possible to study the dynamics of how the ratio and abundance of the descendants of individual cancer cells change both in the case of normal tumor growth and under the influence of various methods of therapy [25,26]. These studies have demonstrated the presence of rare populations of cancer cells that are resistant to therapy. During initial tumor growth, these cells do not have significant advantages in the rate of proliferation, and, therefore, their population is low. However, therapy leads to the death of the bulk of cancer cells, and as a result, this small population of rare cells can form a new tumor.

Any therapeutic effect on a tumor becomes an additional factor providing selective conditions for the survival of cancer cells. In most cases, tumor cells carrying mutations in genes which belong to pathways targeted by the drug, gain selective advantage [27]. The acquisition of therapy resistance can be mediated by an advantage in proliferation of pre-existing clones under the therapy-induced selective conditions. On the other hand, therapy could lead to the emergence and accumulation of new genomic aberrations that provide advantages to the cells carrying them (acquired resistance). It is unclear, which scenario prevails in each case. Research shows that either of these scenarios can be implemented, or possibly both, depending on the type of cancer and the applied therapeutic strategy. Thus, DNA-damaging drugs like alkylating agents have a more mutagenic impact on cells [28]. Chemotherapy of high-grade serous ovarian cancer with platinum-based drugs causes a large number of new somatic mutations in cells [29]. A similar induction of the mutagenesis occurs in glioblastoma under temozolomide therapy [30]. By contrast, combined chemotherapy of bladder cancer with gemcitabine and cisplatin leads to the selection of pre-existing cell populations [31,32]. Likewise, slowly proliferating cells with elevated self-renewal potential can trigger the relapse of acute leukemia after therapy [33,34]. In the case of triple-negative breast cancer, it appears that cells with resistant genotypes had already existed in tumors and were selected by neoadjuvant chemotherapy consisting of epirubicin, docetaxel, and bevacizumab [20].

Particular importance among pre-existing cell populations is attached to a special group of cells with stem properties. A rare population of cancer stem cells is thought to be resistant to therapy and may repopulate the tumor through their self-renewing potential. Such cellular hierarchy models were proposed for several cancers including glioblastoma [35], leukemia [36], breast cancer [37], colorectal cancer [38], etc. However, the definition of cancer stem cells remains highly controversial because of their unstable phenotype [39,40]. Markers used to isolate cancer stem cells are not unique to a given cancer and might define populations of cells in different states. Growing body of evidence suggests that stemness features are associated mainly with intrinsic plasticity of cancer cells and are determined by a dynamic extracellular microenvironment [35,41,42,43]. Novel single-cell expression profiling studies and transcriptome-based lineage trajectory prediction algorithms may provide new insights for the understanding of cancer stem cells properties [35,44].

In addition to understanding which genotypes prone to be adaptively selected in response to chemotherapy, it is equally important to define how such cells evolve further and acquire an increasingly therapy-resistant phenotype. Due to the development of single-cell RNA sequencing technologies, it has become possible to study transcriptional profiles of individual cells and track programs of transcriptional reprogramming induced by therapy.

In the above-mentioned study, Kim et al. used single-cell transcriptome sequencing of triple-negative breast cancer samples to find that transcriptional changes are not initially programmed in tumor cells, but are activated under the effect of neoadjuvant chemotherapy [20]. Thus, it was shown that the expression of genes involved in extracellular matrix degradation, PI3K/AKT/mTOR pathway, angiogenesis, hypoxia signaling pathway, and epithelial-to-mesenchymal transition (EMT) increase in persistent tumor cells in response to chemotherapy.

Analysis of the transcriptional profiles of muscle-invasive urothelial bladder cancer samples before and after tipifarnib treatment made it possible to demonstrate that tumor cells that survived through therapy are in a state of dormancy, also characterized by increased expression levels of the *IGFBP7, MDK,* and *B2M* genes [24]. Such a slow-cycling, persistent quiescent state promotes tumor cell survival during therapy, and such cells can potentially give rise to actively proliferating resistant cells. Thus, B2M can induce EMT via the induction of RAS-independent activation of the PI3K/AKT/mTOR and ERK signaling pathways.

Transcriptional profiling of tumor samples is also important for molecular typing of the tumor and its microenvironment. For example, Izar and colleagues identified 18 separate clusters of malignant and non-malignant cells, differing in their transcriptional signatures in ovarian cancer ascites isolated from patients before and after chemotherapy [22]. Noteworthy, strong differences exist between cells of the same type. Chemotherapy activates the Jak/STAT pathway in some subpopulations of cancer cells and tumor-associated fibroblasts. This indicates the possibility of paracrine and/or autocrine signaling and, as a consequence, co-evolution and remodeling of the tumor environment towards a more aggressive and chemotherapy-resistant phenotype.

Single-cell RNA sequencing of metastatic lung cancer samples demonstrated differences in the transcriptional levels between cancer cells at different points in time: before therapy, in the course of the therapy, when the tumor was either regressing or stable, and upon subsequent progressive disease [21]. It turned out that activation of the WNT/β-catenin pathway in cancer cells contributes to their survival after the initial treatment. As the disease progresses, kynurenine, plasminogen, and gap-junction genes associated with inflammation and carcinogenesis pathways are activated. Besides, as a result of activation of the kynurenine pathway in cancer cells, a noticeable remodeling of the tumor microenvironment occurs, in particular, an antitumor immune response is suppressed.

In a study by Park et al. on a culture of colon cancer cells under the influence of a DNA-damaging drug 5-fluorouracil, the authors identify three unique transcriptome phenotypes and correlate them with the main DNA damage-induced cell-fate responses that include apoptosis, cell cycle arrest, and stress response [23]. In particular, differential regulation of *CDKN1A* or *TP53* genes leads to one or another type of cellular response to therapy.

Most of the studies mentioned above combined single-cell RNA and DNA sequencing, which made it possible to notice that the heterogeneity between the samples at the transcriptome level is lower than at the level of gene mutation. This may suggest that, despite the high diversity of mutations, the transcriptional programs of cancer cells converged on some specific signaling pathways. Thus, using multiple types of cancer, it has been shown that several populations of tumor cells with different states arise after different types of chemotherapy. Therapy can trigger cell death, arrest of cancer cells at a certain phase of the cell cycle, or induce the emergence of a cancer cell population with activated cell injury repair signature due to signaling pathways PI3K/AKT/mTOR, Jak/STAT, WNT/β-catenin, and others. In turn, all of these signaling pathways are known to be capable of inducing EMT, which is often associated with more aggressive tumor behavior [45,46,47].

## 3. Intracellular Mechanisms of Acquired Therapy Resistance

As described above, therapy creates conditions promoting the selection of a pre-existing population of tumor cells. However, numerous studies have shown that the progeny of cells that survived after therapy significantly differs from the parental cells. These changes can occur both at the genome level (new mutations) and at the transcriptome level (gene expression changes which are not associated with DNA mutations) [48] (Figure 1 and Figure 2). Several mechanisms of such de novo adaptation of tumors to the therapy are already well described: regulation of chemotherapeutic drug concentration in the cell, suppression of apoptotic pathways, modification of target proteins, or activation of alternative signaling cascades. Hence, therapy induces intracellular processes allowing the cell to adapt to its action, survive treatment, and subsequently form a new tumor.

For example, the role of drug uptake, efflux, and inactivation in acquired resistance has been demonstrated [49]. Often therapy induces mutations that change the activity or decrease the expression of drug receptors and transporter genes, which ultimately leads to a decrease in the rate of chemotherapeutic drug absorption and development of chemoresistance [50,51]. Besides, the expression of ABC transporters (MDR1, MRP1, MXR, etc.) can increase in response to pharmaceutical drugs, forming the basis for resistance to many other drugs [52,53,54]. Another mechanism is based on the inhibition of proteasomal degradation of drug-target proteins in cancer cells. It has been recently shown that overexpression of disintegrin/metalloproteinase ADAM10 in cancer cells leads to the increased cleavage of JAM-A [55,56]. The cleaved form of JAM-A protein inhibits the proteasomal degradation of HER-2 contributing to the acquired resistance of breast cancer cells to anti-HER2 therapy [55,56].

Also, it has been repeatedly shown that the mutation profile changes significantly during tumor development, and any stress-related conditions, especially chemo- or radiotherapy, enhance mutagenesis due to increased genomic instability and mitotic catastrophes [57]. Thus, in the course of tumor progression and subsequent therapy, additional mutations may occur providing the resistance of tumor cells to the drugs. Among chemotherapy-induced mutations, mutations in genes of the EGFR-dependent signaling pathway (*EGFR*, *HER2*, *RAS*) targeted by the drugs are common [58,59,60,61]. In addition to mutations that lead to a decrease in the effectiveness of targeted therapy, some mutations decrease the effects of less specific chemotherapeutic drugs through the regulation of DNA repair or inhibition of apoptosis. Some of the most common examples are mutations in the *BRCA1* and *BRCA2* tumor suppressor genes [62,63]. Mutations in these genes lead not only to the enhanced probability of genomic instability but also to a greater sensitivity of tumor cells to DNA-damaging agents of the platinum group [64,65] and inhibitors of poly(ADP-ribose) polymerase [63]. Thus, repeated or secondary mutations can restore the functionality of genes and lead to the emergence of resistance to the types of ongoing treatment [62,66,67]. Mutations in the *TP53* gene induced by chemotherapy can lead to an acquisition of resistance to the drugs used [68,69]. In addition to genetic aberrations, epigenetic changes may occur in tumor cells in response to therapy, for example, the silencing of key tumor suppressor genes mediates by DNA hypermethylation [70,71], histone modifications, and chromatin remodeling [72].

However, current observations of drug resistance acquisition cannot be explained solely by genetic and epigenetic aberrations [73]. Many mechanisms require more detailed studies since their contribution to the development of chemoresistance of tumor cells is ambiguous. For instance, the role of EMT in the induction of drug resistance is still not obvious. Moreover, many non-specific drugs effectively act on tumor cells in a specific phase of the cell cycle. However, due to the lack of synchronization of cancer cells at the time of therapy, it is difficult to predict how a particular drug will affect the survival of tumor cells. There is also no consensus on the role of autophagy in the acquisition of drug resistance by cancer cells.

### 3.1. Aspects of Epithelial-to-Mesenchymal Transition

It is generally accepted the mesenchymal tumor phenotype is more aggressive and invasive than the epithelial one, and the epithelial-to-mesenchymal transition (EMT) is often associated with the emergence of resistance to anticancer therapy [74,75]. However, it is still unclear whether the epithelial-to-mesenchymal transition is itself the cause of drug resistance or merely accompanies the processes that induce therapy resistance.

Correlation between the degree of sensitivity of tumor cells to the drug and the expression of genes responsible for a particular phenotype is frequently used to assess the relationship between EMT and chemoresistance. For example, it has been shown that the sensitivity of liver cancer cell lines to cisplatin, gemcitabine, and 5-fluorouracil is associated with decreased expression of E-cadherin and increased expression of mesenchymal transcription factor ZEB1, which inhibits E-cadherin expression [76]. Increased production of a transcription factor FOXC2 is one of the major differences between cisplatin-sensitive and cisplatin-resistant A549 lung cancer cell lines [77]. Similar conclusions were made after investigation of the expression level of genes responsible for EMT in tumor tissue samples from patients and primary cell cultures of glioblastoma [78], prostate cancer [79], breast cancer [80], or non-small cell lung carcinoma [81].

ZEB1 is one of the main transcription factors responsible for the epithelial-to-mesenchymal transition. ZEB1 is activated in response to external stimuli that trigger intracellular signaling cascades PI3K/AKT/mTOR, RAS/ERK, WNT/β-catenin, and NF-κB and inhibits the expression of genes specific for the epithelial phenotype: *E-cadherin, ESRP1, EpCAM, ER-α, RAB25*, and *ST14* [82]. Knockdown of ZEB1 in breast cancer, non-small cell lung carcinoma, and osteosarcoma cell lines leads to an increase in the sensitivity to radiotherapy while its ectopic expression in cell models of breast cancer decreases the sensitivity [83,84,85]. However, it should be noted transcription factors often regulate more than one process. For example, in addition to the induction of EMT, ZEB1 and other EMT transcription factors (SNAIL, SLUG, TWIST) are involved in the regulation of the cell cycle, proliferation, DNA repair, lipid metabolism, pumping chemotherapeutic drugs out of the cell, and activation of T lymphocytes [83,86,87,88,89,90]. Thus, EMT transcription factors participate in a wide range of processes that cause therapy resistance of tumor cells. Moreover, it has been shown that ZEB1 can induce resistance to radiotherapy without induction of EMT [83,84].

Moreover, epithelial phenotype can be reduced post-transcriptionally in several cancer cell lines through re-localization of epithelial proteins from the cell surface to endocytic complexes without activation EMT transcription factors [91]. This process may provide recycling of epithelial proteins back to the cell surface and might partially explain the existence of hybrid epithelial/mesenchymal state of cancer cells. It is noteworthy that EMT in cancer cells, both in vitro and in vivo, occurs through a range of distinct intermediate epithelial/mesenchymal state with various invasion and differentiation characteristics [92,93]. Moreover, cancer cells existing in this hybrid state were shown to be more aggressive than cells with a complete mesenchymal phenotype [92,94]. In addition, it should be borne in mind that EMT activation programs may differ depending on the type of tissue as well as the specific tumor microenvironment [92,95]. For example, the activation of transcriptional factors SNAI1 and TWIST1 induces dissemination of breast cancer cells, while both of these factors are dispensable for metastasis but induces chemoresistance in pancreatic cancer [96,97,98]. Besides transcriptional control, different regulatory networks such as epigenetic modifications, alternative splicing, and protein stability further complicate understanding of the whole picture of EMT process [75,95,99,100,101].

The EMT is directly related to metastasis and invasion of tumor cells. In the model of breast cancer, Hausser’s group shows that the main processes necessary for tumor progression, i.e., cell division, maintenance or increase in biomass and energy, lipogenesis, immune interactions, invasion, and tissue remodeling, cannot occur simultaneously [102]. Other groups of researchers also notice the difficulty of simultaneous processes of proliferation and invasion [103,104,105]. Hence, a tumor cell has to choose whether to maintain its metabolism on glucose or lipids, actively proliferate, avoid an immune response, or metastasize [102]. As will be discussed below, proliferative activity and being at a certain stage of the cell cycle can increase or decrease the sensitivity of tumor cells to the effects of therapy. For example, metastatic tumor cells can be more resistant to DNA-damaging drugs or to agents that stabilize microtubules.

Thus, signals that activate EMT often also trigger other processes associated with the emergence of therapy resistance of tumor cells. Therefore, it is often impossible to isolate the effects caused by EMT alone. Nevertheless, changes in the EMT markers in tumor cells can be used to assess the success of anticancer therapy.

### 3.2. Cell Cycle-Mediated Chemoresistance of Cancer Cells

Many chemotherapeutic drugs affect the cell in a certain phase of the cell cycle. Drugs that directly or indirectly damage DNA (cisplatin, doxorubicin, 5-fluorouracil, or topotecan) are active mainly in the S phase of the cell cycle [106]. Drugs leading to improper assembly of the spindle apparatus—vinca alkaloids and taxanes (docetaxel and paclitaxel)—are the most toxic to cells in the M phase [107].

**Figure 3:**
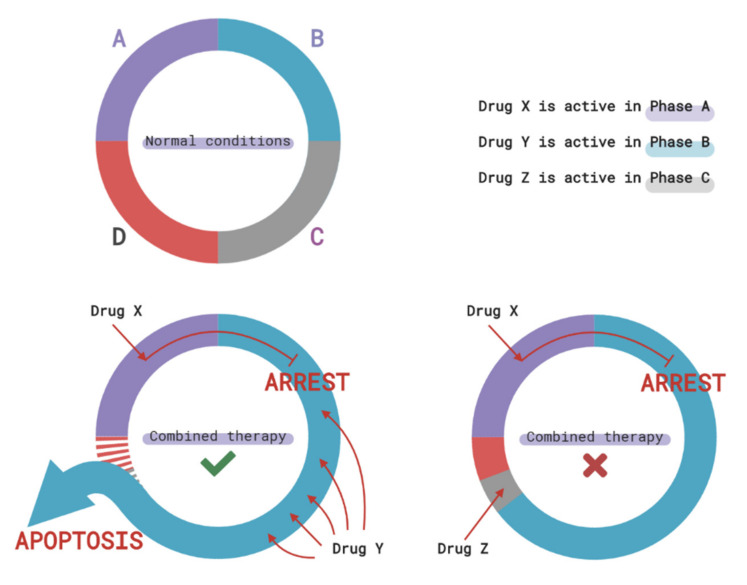
The success of combined therapy depends on the cell cycle. The use of chemotherapeutic drugs that depend on the phase of the cell cycle and cause its arrest may lead to both higher and lower effectiveness of the combined therapy. Arresting the cell cycle in a phase corresponding to the maximum effectiveness of the second drug prolongs its time of action. Arresting the cell cycle in a phase preceding the action of the second drug impairs its effects. A, B, C, and D represent 4 phases of the cell cycle.

The arrest of the cell cycle in the phase preceding the phase of exposure to the chemotherapeutic drug can reduce the effectiveness of treatment (Figure 3). For example, the arrest of the cell cycle in the G1-phase leads to the emergence of resistance of melanoma cells to bortezomib (protease inhibitor) and temozolomide (alkylating agent). Therefore, pretreatment of melanoma cells with drugs that cause cell cycle arrest in the G1 phase (for example, an inhibitor of the MAPK pathway) can lead to the emergence of resistance to bortezomib and temozolomide, but treatment in the reverse order does not lead to the development of resistance [108]. Resveratrol provokes cell cycle arrest in the S phase which leads to a delayed entry of the cell into mitosis. This treatment reduces the effectiveness of an M phase-specific chemotherapeutic drug paclitaxel but enhances the efficacy of cisplatin acting in the S phase [109]. Synchronization of cells in the M phase increases the sensitivity of ovarian cancer cells to paclitaxel by 2–3 times [110]. Additionally, hypoxia-induced arrest in the G1 phase increases the resistance of oral cancer to 5-fluorouracil 10-fold [111].

Furthermore, the arrest of the cell cycle at a certain phase gives the damaged cell the opportunity to recover before it enters mitosis, while premature division can trigger apoptosis. This phenomenon is most pronounced for DNA damaging agents. For example, cisplatin causes replicative stress, which activates the phosphokinases ATR, CHK1, and WEE1, thereby preventing further course of the cell cycle and leading to the arrest of tumor cells in the S phase. A reduced level of WEE1 entails a premature start of mitosis in cancer cells that carry severe DNA damage and their subsequent death [112]. Another group of authors showed that the different resistance of triple-negative breast cancer cell lines to cisplatin was associated with different mechanisms of cell cycle regulation in response to stress, rather than alteration in the DNA repair system [73]. Thus, resistant breast cancer cell lines, in contrast to sensitive ones, enter mitosis only after complete repair of DNA damaged by cisplatin. G3BP2, HMMR, and NEK2 have been proposed as participants in the regulation of the cell cycle that determine the fate of the cell in response to cisplatin.

However, there is an opposite view on the matter: the prolonged arrest of the cell cycle may as well trigger apoptosis. In the model of cisplatin-sensitive lung cancer cell line A549 and its cisplatin-resistant modification, it was shown that resistant cells tend to avoid G2/M arrest of the cell cycle [113,114]. Moreover, a resistant analog of breast cancer cell line MDA-MB-231 maintained a high level of expression of genes responsible for the progress through the cell cycle even after treatment with doxorubicin, while corresponding sensitive cells were arrested in the sub-G1 phase and undergone apoptosis [115].

Thus, the cell cycle phase of tumor cells treated with chemotherapeutic drugs can play an important role both during the direct action of chemotherapy and during cell response to it. Understanding the peculiarities of cell cycle regulation in tumor cells, as well as the effect of chemotherapeutic drugs on the cell cycle in combined chemotherapy, can be used to increase the efficiency, as well as to develop new treatment strategies.

### 3.3. Autophagy as a Way to Avoid Therapy-Induced Cell Death

Autophagy is present at a basal level in all cell types and serves to maintain intracellular homeostasis by recycling molecules and entire organelles within specialized membrane structures [116]. It plays a controversial role in tumor progression. At the initial stages of oncogenesis, autophagy suppresses tumor development by preventing the accumulation of damaged proteins and organelles, followed by inhibition of inflammation and oxidative stress, and a contribution to oncogene-induced senescence [117,118]. At later stages of tumorigenesis, it stimulates the survival of tumor cells by reducing stress levels during hypoxia and nutrient starvation [117,118]. However, prolonged activation of autophagy in cancer cells, as well as exposure to chemotherapeutic drugs, can lead to autophagic cell death due to gradual self-degradation [119,120,121,122].

Chemotherapy-induced stress can trigger both autophagy and apoptosis, the balance between these processes is highly dynamic, and can be controlled by the p38-MAPK, JNK, and p62 pathways [123,124]. The prevalence of autophagy over apoptosis is often associated with the resistance of tumor cells [125,126].

Activation of apoptosis and autophagy under the influence of therapy can happen simultaneously, sequentially, independently, synergistically, or antagonistically of one another [127]. In some cases, the protective autophagy pathway can be activated, and its inhibition increases therapy effectiveness. For example, activation of apoptosis induced by capsaicin or epirubicin is possible only after the blockade of protective autophagy [128,129]. Thus, successful combinations of drugs that simultaneously trigger the apoptosis of tumor cells and inhibit autophagy have been demonstrated [126,130,131,132,133,134,135,136,137,138,139,140,141,142]. However, therapeutic inhibition of autophagy may also induce the opposite effect. For example, inhibition of autophagy during the treatment with resveratrol and feroniellin A leads to the suspension of tumor cell death, demonstrating the pro-apoptotic role of autophagy in oncogenesis [143,144].

In addition to the above mentioned, multiple chemotherapy drugs that stimulate autophagy also affect PI3K/Akt/mTOR, AMPK, ERK, and JNK pathways, thereby promoting cancer cell survival [145,146,147,148,149]. Hypoxia can play a role in the activation of protective autophagy. For example, the antiangiogenic agent bevacizumab induces hypoxic stress and triggers autophagy through the HIF-1α/AMPK pathway, stimulating the survival of glioblastoma cells [150]. Treatment of cancer cells with cisplatin or taxol under hypoxic conditions induces protective autophagy, which promotes stress elimination and ensures the survival of lung cancer and breast cancer cells, respectively [146,151]. MicroRNAs may also be responsible for the activation of protective autophagy when exposed to chemotherapy. For example, cisplatin leads to a decrease in the level of miR-199a-5p in hepatocellular carcinoma cells, which, as a result, stimulates the activation of autophagy and promotes the proliferation of tumor cells [152].

Summing up, the effect of autophagy during treatment with various chemotherapeutic drugs is not predictable and depends on many factors, including the type of cancer. For example, exposure to metformin promoted the myeloma cell death by activating autophagy but led to the survival of breast cancer cells by stimulating protective autophagy [153,154]. Several studies have shown contradictory results upon autophagy inhibition in conjunction with the effect of sorafenib on human hepatocellular carcinoma cells [155,156]. The stage of autophagy is also should be taken into account. For example, in the case of treatment of glioma cells with imatinib, the cytotoxicity of the chemotherapeutic drug increased with inhibition of late stage of autophagy and decreased with inhibition of early stage of autophagy. Presumably, the increased cytotoxicity may be related to the number of sequestered mitochondria that are ineffectively eliminated and provoke additional stress [157].

## 4. Contribution of Intercellular Communication to Acquired Therapy Resistance

**Figure 4:**
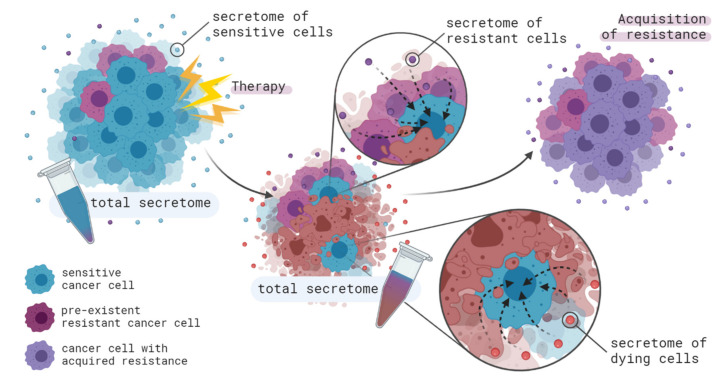
Features of tumor intercellular communication in response to therapy. During therapy, most of the sensitive tumor cells die, which leads to a decrease of their fraction in the tumor population and an increase in the proportion of resistant clones. Similarly, the composition of the total cell secretome and, consequently, its ability to participate in the process of acquiring therapy resistance change. Moreover, tumor cells dying under the effect of therapy release a number of molecules into the extracellular space which can induce the acquisition of resistance in untreated tumor cells.

Two mechanisms of therapy resistance development in cancer were described above (clonal selection and intracellular changes induced by therapy). Both of them occur at the level of individual cells. However, there are also more complex mechanisms based on the interaction of various populations of cells with each other. These mechanisms have been first demonstrated less than 10 years ago and some of them are rather poorly understood, however, they could be described by the following general scheme. The therapy affects one cell population, and these cells secrete a variety of molecules, both in free form and encapsulated within extracellular vesicles. These secreted components, in turn, can induce the development of resistance in surrounding populations of cells. Thus, it is important to understand that tumor cells function within the tumor microenvironment: dying and surviving cells constantly exchange information with each other and with cells of the tumor stroma (Figure 1 and Figure 4). There are several ways to conduct the intercellular communication, namely directly (through cell junctions, receptor–ligand binding, or tunneling nanotubes) and indirectly (through signaling with extracellular vesicles or soluble molecules) [158]. Most often, in the literature on the acquisition of therapy resistance, the concept of intercellular communication means precisely the secretion and uptake of signaling molecules both in free form and via extracellular vesicles. Therefore, we will focus our attention on this process.

### 4.1. From Cancer Cells to Cancer Cells

Chemotherapy, especially preoperative chemotherapy, induces a significant decrease in tumor size, which implies massive cell death. During the process, most of the sensitive tumor cells die, but for some reason, e.g., lack of proliferative activity, uneven distribution of the drug in the tissue, etc., some sensitive tumor cells can survive and continue to exist after treatment. Thus, several patterns of tumor cell secretion after chemotherapy can be distinguished: (i) secretion by sensitive or resistant clones that were not affected by chemotherapy; (ii) secretion by dying tumor cells; (iii) secretion by tumor cells that have progressed into a state of senescence (a durable form of growth arrest) (Figure 4).

Therapy-sensitive and therapy-resistant tumor cells are known to secrete significantly different sets of signaling molecules [159,160,161,162,163,164,165]. Using cell models of breast cancer [166,167,168,169,170,171], ovarian cancer [172,173], prostate cancer [174], melanoma [175,176], acute lymphoblastic leukemia [177], acute myeloid leukemia [178], colorectal cancer [179], esophageal cancer [180], non-small cell lung cancer [181], osteosarcoma [182], and renal cell carcinoma [183] therapy-resistant tumor cells were shown to release a number of molecules into the extracellular space, which contributes to the acquisition of resistance in more sensitive tumor cells.

One of the components frequently involved in such intercellular communication is microRNAs: miR-30a and miR-100 [166]; miR-19b and miR-20a [178]; miR-155 [171]; miR-221 [167]; miR-222 [166,167,170,181]. Proteins ALKres [176], GSTP1, p-STAT3 [179], PDGFRb [175], UCH-L1 [169], Syntaxin 6 [184], and EphA2 [185] also play an important role. The exact mechanisms of action of most of these effector molecules have not yet been studied. However, many of them facilitate the avoidance of cell death, a decrease in the expression of targets of targeted therapy, either directly or through changes in the regulation of the cell cycle, EMT, autophagy.

The phenomenon of the ABC transporters transfer from resistant tumor clones to more sensitive ones was demonstrated by multiple studies. It was shown that multidrug resistance proteins, as well as their transcripts, can be exported by chemoresistant cells and absorbed by sensitive tumor cells as parts of extracellular vesicles, thereby contributing to a reduction of the chemotherapeutic drug concentration inside the recipient cell and a decrease its sensitivity to therapy [168,169,173,177,178,182].

During therapy, an abundance of resistant clones grows within the tumor, which inevitably leads to an increase in the content of the molecules secreted by them in the total tumor secretome. As a result, the remaining sensitive clones acquire resistance to therapy during communication with the increasing number of resistant tumor cells.

In addition to the transfer of individual molecules and vesicles, the possibility of intercellular transport of large structures such as mitochondria was demonstrated both during the communication of tumor cells with their microenvironment (bone marrow mesenchymal stem cells, cancer-associated fibroblasts, immune cells) and within the tumor cell population itself [186,187,188,189]. Tumor cells that have damaged mitochondria (for example, as a result of therapy) receive healthy mitochondria from donor cells, thereby restoring their mitochondrial activity. On the other hand, the release of mitochondria by tumor cells leads to the modification of the surrounding “healthy” stromal cells [190].

Apparently, the most significant contribution to intercellular communication is made by components secreted from tumor cells dying under the effect of chemotherapy, since the first stages of chemotherapy induce death of a major part of the tumor cell population. The death of a significant part of the tumor cells occurs. It turns out that dying tumor cells are capable of releasing components into the extracellular space that “prepare” intact tumor cells for subsequent therapeutic insults [31,191,192,193].

After treatment with tyrosine kinase inhibitors, dying sensitive melanoma and lung cancer cells secrete into the extracellular space components that stimulate chemoresistance and metastasis of recipient tumor cells due to activation of the PI3K/AKT/mTOR pathway [191]. Moreover, recipient cancer cells acquire therapy resistance only in the presence of a large fraction of dying sensitive tumor cells nearby. However, specific signaling molecules provoking these events in resistant clones have not been identified.

Dying tumor cells of breast cancer and bladder cancer secrete prostaglandin E2 (PGE2) and arachidonic acid, causing the repopulation of residual tumors through activation of the WNT/β-catenin pathway and stimulation of EMT [31,193]. The increased release of PGE2 and arachidonic acid was due to the activation of calcium-independent phospholipase A2 (iPLA2) by activated caspase 3 in dying tumor cells.

A significant number of glioblastoma cells dying after radiotherapy stimulates the acquisition of a more aggressive phenotype by surviving cancer cells by secreting apoptotic vesicles containing various spliceosomal proteins [192]. These vesicles induce changes in the splicing of mRNAs encoding key proteins associated with EMT and cell cycle regulation; they also upregulate glycolysis in recipient tumor cells. In particular, the splicing factor RBM11 can be transferred via apoptotic vesicles and regulate the cell cycle of recipient cells through binding to mRNAs that encode Cyclin D1 (cell cycle regulator) and MDM4 (apoptosis regulator) and switching their splicing to more oncogenic isoforms cyclin D1a and MDM4s, respectively, that were associated with a poorer prognosis for patients.

Noteworthy, various types of anticancer therapy: radiotherapy [192,194], alkylating agents [195,196], topoisomerase inhibitors [197], antimetabolites [198,199], and taxanes [200,201], as well as kinase inhibitors [191,197] trigger such communication between tumor cells. Components of the spliceosome [192]; PGE2 [31,193]; survivin [200,202]; MDR1 [203], HMGB1 [204,205]; several microRNAs (miR-21 [196], miR-155 [198,199], and miR-194-5p [194], and lincRNA-VLDLR [197] have been proposed as the main participants that trigger the acquisition of therapy resistance by tumor cells (Figure 2).

Unfortunately, there are only a few published studies that analyze global differences in the profiles of tumor cell secretion before and after chemotherapy. In this regard, it is still difficult to identify main effector molecules that can change the state of recipient tumor cells towards a more aggressive phenotype. The situation is further complicated by the fact that the secretion profiles of tumor cells that die through different mechanisms also vary significantly [206]. Thus, cells that die by necroptosis release a significant amount of lysosomal proteins, while apoptotic cells secrete histones and other components of the nuclear fraction into the extracellular space.

Also, under the effect of chemotherapy, tumor cells can transform into a state of irreversible or transient senescence. Cells with a senescence-associated secretory phenotype (SASP) secrete a special set of molecules that promotes the emergence of drug resistance in chemonaïve recipient tumor cells. This phenomenon has been demonstrated in senescent malignant pleural mesothelioma, melanoma, and breast cancer cells [207,208,209,210,211].

Thus, the components secreted by tumor cells can significantly destabilize the homeostasis present in chemonaïve tumor cells and provoke cell transition from less resistant to a more resistant state.

### 4.2. Communication between Cancer Cells and Surrounding Stromal Cells

Tumor microenvironment (TME) includes tumor-associated fibroblasts (CAFs), immune cells, mesenchymal stem cells, adipocytes, and endothelial cells. All of them can participate in both maintenance and inhibition of tumor growth [158,212,213]. The contribution of stromal cells to the development of therapy resistance differs from patient to patient since their abundance can vary over a very wide range, even among the same type of tumor [214]. Moreover, the proportion of stromal cells can be changed during treatment, thereby affecting the course of therapy. Thus, a number of studies have shown that a high content of stromal cells may indicate poor prognosis of patient outcome [215].

Cancer cells actively remodel the tumor microenvironment during therapy. For example, cells that die from chemotherapy release molecules that regulate the immune response, stimulate angiogenesis, alter the physicochemical parameters of the tumor microenvironment, or activate cellular invasion [216]. An immunosuppressive effect has been shown for molecules released by dying cancer cells, e.g., secreted CCL20 recruits regulatory T cells via the FOXO1/CEBPB/NF-κB signaling [217]; sphingosine-1 phosphate activates and polarizes of tumor-associated macrophages into M2 macrophages. These M2 macrophages secrete anti-inflammatory IL-10, and PGE2 supporting the migration of endothelial cells and angiogenesis [218]. In addition to promoting angiogenesis, PGE2 released from dying cells also provokes the recruitment of macrophages, CAFs, and neutrophils into TME and suppresses the antitumor functions of T cells and natural killer cells [219].

The major component of the tumor stroma is tumor-associated fibroblasts [220]. Communication between CAFs and tumor cells promotes the formation of a therapy-resistant phenotype in the latter [221]. For example, CAFs have been associated with the development of resistance to gemcitabine in pancreatic cancer through secretion of miRNA-106b [222]; to gefitinib in non-small cell lung cancer via secretion of IGF-1 and HGF [223]; to 5-fluorouracil in colorectal cancer [224]; to cisplatin in esophageal squamous cell cancer through secretion of PAI-1 [225] and TGFβ1 [226], in gastric cancer through secretion of miR-522 [227], IL-11, IL-6, and other cytokines and growth factors [228], and in lung adenocarcinoma due to secretion of IL-11 [229].

In response to therapy treatment, CAFs secrete microRNAs encapsulated into extracellular vesicles. After entering recipient tumor cells, miRNA-106b or miR-522 reduce cancer cells’ sensitivity to the chemotherapy by targeting antiproliferative and pro-apoptotic protein TP53INP1 [222] and arachidonate lipoxygenase (ALOX15), leading to a decrease in the accumulation of lipid peroxides in cells and inhibition of ferroptosis [227]. The mechanism of action of miR-21 is based on targeting APAF1, which leads to impairment in the activation of caspase-3 and apoptosis [230]. Similarly, miR-92a-3p activates the Wnt/β-catenin pathway and directly inhibits pro-apoptotic proteins FBXW7 and MOAP1 [231]. Proteins secreted by CAFs during chemotherapy also contribute to increased proliferation of recipient tumor cells and the formation of a more resistant phenotype therein. Thus, exosomal Wnts stimulate the dedifferentiation of cancer cells directly through Wnt signaling [232]. Secreted PAI-1 activates the AKT and ERK1/2 signaling pathways and inhibits caspase-3 activity [225]. Under the influence of HGF and IGF-1 secreted by CAFs, tumor cells increase the expression of Annexin A2, which may lead to the induction of EMT [223]. As a result of the secretion of IL-11 by CAFs, STAT3 is phosphorylated and increases of expression of anti-apoptotic proteins Bcl-2 and survivin in cancer cells [229]; IL-8 activates NF-κB and elevates the expression of MDR1 [233]. Also, TGFβ1 [226], IL-6 [234,235], and GDNF [236] participate in CAF-mediated chemoresistance. Special attention should be paid to the fact that some of the above-mentioned molecules, namely miR-106b [222], miR-522 [227], IL-11 [229], PAI-1 [225], and GDNF [236] are secreted by CAFs in response to therapy.

Not surprisingly that cancer therapy leads to tissue damage and, as a consequence, the attraction and accumulation of a large number of myeloid cells, mainly tumor-associated macrophages (TAM), to the damaged areas, where they participate in the restoration of tumor tissues [237]. There are various mechanisms through which the process occurs: suppression of the T-cell immune response, activation of the revascularization process, and inhibition of cell death signaling pathways in cancer cells by secretion of various growth factors, chemokines, and cytokines. For example, decreased expression of miR155-5p has been shown to increase the expression of C/EBPβ and IL6 in TAMs, which in turn leads to activation of the IL6R/STAT3/miR-204-5p pathway and induction of chemoresistance in colorectal cancer cells [238]. Similarly, in pancreatic ductal adenocarcinoma, macrophages that phagocytose apoptotic cells secrete the 14-3-3 zeta/delta (14-3-3ζ) protein, which inhibits apoptosis through 14-3-3ζ/Axl pathway, leading to phosphorylation of Akt and activation of cellular pro-survival mechanisms in the tumor cells [239]. The role of tumor-associated macrophages in the development of chemoresistance was discussed in detail in a recent review by Larionova et al. [240].

The presence of mesenchymal stem cells (MSC) in tumors increases in response to chemotherapy. In particular, it has been shown that after gemcitabine treatment of pancreatic adenocarcinoma, MSCs begin to actively secrete CXCL10 and activate the CXCR3 signaling in cancer cells and thus contribute to drug resistance and tumor regrowth [241]. A similar mechanism has been demonstrated for gastric cancer: TGF-β1 secreted by MSCs activates SMAD2/3 and thereby induces the expression of lncRNA MACC1-AS1 in tumor cells, which promotes fatty acid oxidation-dependent stemness and chemoresistance through antagonizing miR-145- 5p [242].

The considered examples of crosstalk between cancer cells and cells of the tumor microenvironment allow us to conclude that therapy leads not only to a change in the cellular composition of the tumor microenvironment, but also to a change in the secretion profiles of its cells. Together, these mechanisms contribute to the acquisition of resistance to the applied therapy.

## 5. Conclusions

The results of cancer research using high throughput single-cell technologies, including single-cell DNA and RNA sequencing, lead to a more comprehensive understanding of tumor heterogeneity, complexity, and high plasticity. It is important to study mutations, copy number alterations, epigenetic changes, and gene expression profiles to understand how different populations of malignant and non-malignant cells within a tumor can potentially respond to therapy (in pre-treatment samples), or how cells that survive after therapy become more resistant (in samples after treatment). Knowing which therapy-resistant phenotypes are present in a newly emerging tumor will allow physicians to choose the best strategies to avoid drug resistance or re-sensitize tumor cells.

The emergence of the first cytotoxic antineoplastic drugs (DNA alkylating agents, antimetabolites, antimitotics, topoisomerase inhibitors, cytotoxic antibiotics, etc.) marks a milestone in the history of anticancer therapy [243]. Although these drugs are not specific for cell types or targets, they are still the standard of care for many types of cancer (e.g., acute leukemias, breast cancer, ovarian cancer, colorectal cancer, lung cancer, glioblastoma). However, it quickly became clear that the stress response mechanisms existing in a cancer cell allow them to overcome the impairments that arise upon exposure to these classes of chemotherapy. Since the 1980s, more specific drugs have begun to appear, such as selective kinase inhibitors and monoclonal antibodies. These classes of therapy target specific molecules, such as EGFR, VEGF, PD1, HER2, mTOR, etc. Their use in combination with traditional chemotherapeutic drugs has allowed to achieve significant progress in the treatment of advanced and/or metastatic cancers. However, the cells still manage to adapt to targeted therapy or combined treatment regimens by activating alternative signaling pathways.

In the case of cancers with an extremely high relapse rate, for which the current standard of care demonstrates no substantial survival benefit (glioblastoma, ovarian cancer, etc.), it might be promising to use drugs that can alter intercellular communication. Thus, compounds that activate interactions between the tumor and the patient’s immune cells have shown their high efficiency [244,245]. Also, drugs that inhibit molecular mediators of intercellular communication are currently being actively investigated [246,247]. Therefore, future therapeutic developments should take into account the highly dynamic heterogeneity and the complexity of the microenvironment of tumor cells.

## Abbreviations

14-3-3ζ	14-3-3 protein zeta/delta
ADAM10	Disintegrin and metalloproteinase domain-containing protein 1
AKT	Protein kinase B
ALK	ALK tyrosine kinase receptor
ALOX15	Polyunsaturated fatty acid lipoxygenase ALOX15
AMPK	AMP-activated protein kinase
APAF1	Apoptotic protease-activating factor 1
ATR	Serine/threonine-protein kinase ATR
Axl	Tyrosine-protein kinase receptor UFO
B2M	Beta-2-microglobulin
Bcl	B-cell lymphoma protein
BRCA1/2	Breast cancer type 1/2 susceptibility protein
CAFs	Cancer-associated fibroblasts
CCL20	C-C motif chemokine 20
CDKN1A	Cyclin-dependent kinase inhibitor 1
CEBPB	CCAAT/enhancer-binding protein beta
CHK1	Serine/threonine-protein kinase Chk1
CXCL10	C-X-C motif chemokine 10
CXCR3	C-X-C chemokine receptor type 3
EGFR	Epidermal growth factor receptor
EMT	Epithelial-to-mesenchymal transition
EpCAM	Epithelial cell adhesion molecule
EphA2	Ephrin type-A receptor 2
ERK1/2	Mitogen-activated protein kinase 3/1
ER-α	Estrogen receptor alpha
ESRP1	Epithelial splicing regulatory protein 1
FBXW7	F-box/WD repeat-containing protein 7
FOXC2	Forkhead box protein C2
FOXO1	Forkhead box protein O1
G3BP2	Ras GTPase-activating protein-binding protein 2
GDNF	Glial cell line-derived neurotrophic factor
GSTP1	Glutathione S-transferase P
HER2	Receptor tyrosine-protein kinase erbB-2
HGF	Hepatocyte growth factor
HIF-1α	Hypoxia-inducible factor 1-alpha
HMGB1	High mobility group protein B1
HMMR	Hyaluronan mediated motility receptor
IGF-1	Insulin-like growth factor I
IGFBP7	Insulin-like growth factor-binding protein 7
Ils	Interleukins
iPLA2	Calcium independent phospholipase A2
Jak	Tyrosine-protein kinase JAK
JAM-A	Junctional adhesion molecule A
JNK	c-Jun N-terminal kinase
lncRNAs	Long non-coding RNAs
MAPK	Mitogen-activated protein kinase
MDK	Midkine
MDM4	Protein Mdm4
MDR1	ATP-dependent translocase ABCB1
miR	microRNA
MOAP1	Modulator of apoptosis 1
MRP1	Multidrug resistance-associated protein 1
MSCs	Mesenchymal stem cells
mTOR	Mammalian target of rapamycin
MXR	Broad substrate specificity ATP-binding cassette transporter ABCG2
MYCN	N-myc proto-oncogene protein
NEK2	Serine/threonine-protein kinase Nek2
NF-κB	Nuclear factor kappa-light-chain-enhancer of activated B cells
p38-MAPK	p38 mitogen-activated protein kinase
p62	Ubiquitin-binding protein p62
PAI-1	Plasminogen activator inhibitor 1
PD1	Programmed cell death-1
PDGFRb	Platelet-derived growth factor receptor beta
PD1L1	Programmed cell death 1 ligand 1
PGE2	Prostaglandin E2
PI3K	Phosphoinositide 3-kinase
p-STAT3	Phospho-Stat3, Signal transducer and activator of transcription 3
RAB25	Ras-related protein Rab-25
RAS	Ras GTPase
RBM11	Splicing regulator RBM11
SASP	Senescence-associated secretory phenotype
SLUG	Zinc finger protein SNAI2
SMAD2/3	Mothers against decapentaplegic homolog 2/3
SNAIL	Zinc finger protein SNAI1
ST14	Suppressor of tumorigenicity 14 protein
STAT	Signal transducer and activator of transcription
TAMs	Tumor-associated macrophages
TGFβ1	Transforming growth factor beta-1 proprotein
TME	Tumor microenvironment
TP53	Cellular tumor antigen p53
TP53INP1	Tumor protein p53-inducible nuclear protein 1
TWIST	Twist-related protein
UCH-L1	Ubiquitin carboxyl-terminal hydrolase isozyme L1
UTR	Untranslated region
VEGF	Vascular endothelial growth factor A
WEE1	Wee1-like protein kinase
WNT	Proto-oncogene Wnt
ZEB1	Zinc finger E-box-binding homeobox 1
